# Comparison between the HCV IRES domain IV RNA structure and the Iron Responsive Element

**DOI:** 10.1186/1477-5751-8-4

**Published:** 2009-02-18

**Authors:** Ebenezer Tumban, Jenna M Painter, William B Lott

**Affiliations:** 1 Molecular Biology Program, New Mexico State University, Las Cruces, NM 88003-8001, USA; 2 Department of Chemistry and Biochemistry, New Mexico State University, Las Cruces, NM 88003-8001, USA; 3 Institute for Health and Biomedical Innovation, School of Life Sciences, Queensland University of Technology, Brisbane, QLD 4001, Australia

## Abstract

**Background:**

Serum ferritin and hepatic iron concentrations are frequently elevated in patients who are chronically infected with the hepatitis C virus (HCV), and hepatic iron concentration has been used to predict response to interferon therapy, but these correlations are not well understood. The HCV genome contains an RNA structure resembling an iron responsive element (IRE) in its internal ribosome entry site (IRES) structural domain IV (dIV). An IRE is a stem loop structure used to control the expression of eukaryotic proteins involved in iron homeostasis by either inhibiting ribosomal binding or protecting the mRNA from nuclease degradation. The HCV structure, located within the binding site of the 40S ribosomal subunit, might function as an authentic IRE or by an IRE-like mechanism.

**Results:**

Electrophoretic mobility shift assays showed that the HCV IRES domain IV structure does not interact with the iron regulatory protein 1 (IRP1) *in vitro*. Systematic HCV IRES RNA mutagenesis suggested that IRP1 cannot accommodate the shape of the wild type HCV IRES dIV RNA structure.

**Conclusion:**

The HCV IRES dIV RNA structure is not an authentic IRE. The possibility that this RNA structure is responsible for the observed correlations between intracellular iron concentration and HCV infection parameters through an IRE-*like *mechanism in response to some other cellular signal remains to be tested.

## Background

Hepatitis C virus (HCV) is a positive-sense single-stranded RNA virus that infects about 1% of the world's population [1]. Fifty percent of acute infections progress to chronic liver infection and can lead to cirrhosis of the liver and hepatocellular carcinoma [2,3]. A correlation between chronic HCV infection and intracellular iron homeostasis has been empirically established, but the mechanism of this relationship is not understood [4,5]. Increased intracellular iron concentration has been shown to enhance HCV IRES-dependent translation, and two cellular factors, p85 and p110, bind to both the HCV internal ribosome entry site (IRES) and the iron responsive element (IRE) in an iron-dependent manner [6]. Serum ferritin and hepatic iron concentrations are frequently elevated in chronically infected patients [7], and hepatic iron concentration has been used as a predictor of response to interferon therapy [8].

An RNA structural element located at the junction between the open reading frame (ORF) and the 5' untranslated region (UTR) of the HCV genome bears striking structural and sequential similarities to the iron responsive element (IRE), an RNA structure found in some eukaryotic mRNA that controls gene expression in response to intracellular iron concentration [9]. If this HCV RNA element functions as an authentic IRE, then iron depletion in an HCV infected cell would be expected to specifically inhibit viral protein expression and could potentially decrease subsequent viral replication. A possible relationship between this HCV RNA structure and the IRE was investigated.

**Figure 1:**
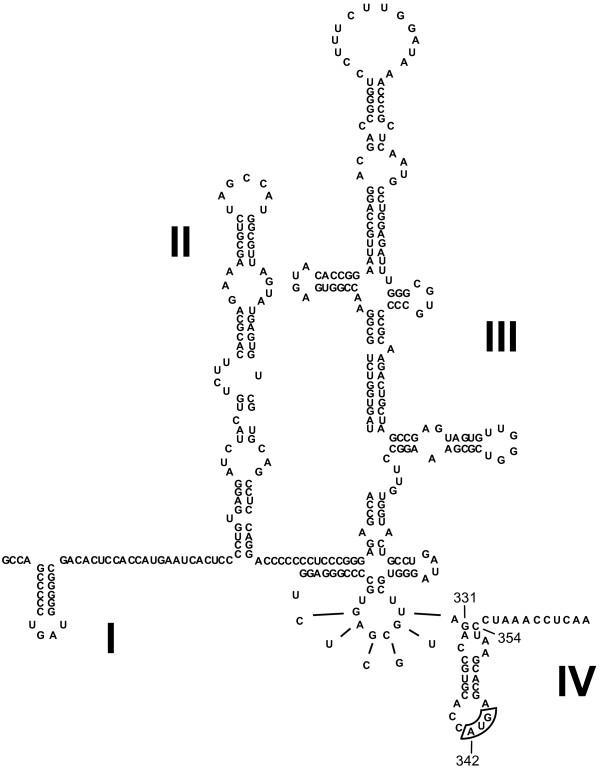
**The HCV 5'UTR secondary structure**. The base pairing convention described by Honda *et al *[17] is used to depict the predicted base pairing in the HCV IRES RNA structure for genotype 1b. The structural domains are labelled I-IV. The authentic HCV start codon at HCV nt 342–344 is boxed. The wild type HCV IRES dIV RNA sequence used in this study represents HCV nt 331–354.

HCV belongs to the *Hepacivirus *genus of the *Flaviviridae *family [10]. The RNA genome is approximately 9.6 kb and serves as a template for both translation and replication. The single long ORF is flanked at the 5' and 3' ends by highly structured UTRs, which are essential for initiation of translation and replication, respectively [11]. The hepacivirus and pestivirus genera of *Flaviviridae *initiate translation via virtually identical non-scanning cap-independent mechanisms that utilize an internal ribosome entry site (IRES) to recruit and assemble the ribosome directly at the initiation site [12-15]. The HCV IRES (figure 1) is a complex and highly conserved RNA structure that is located predominantly within 5' UTR, but is believed to extend into the 5' proximal region of the ORF [16]. It is canonically divided into four structural domains. Domain I is required for efficient viral replication and is not required for viral translation. Domains II and III are necessary and sufficient to recruit and assemble the ribosome at the start site [13]. The domain IV (dIV) RNA structure, which is not essential for efficient translation [17] and is unique to hepaciviruses, has no known function. The pestivirus IRES contains structural domains that are analogous to HCV IRES domains I-III, but lacks domain IV. The border between the 5' UTR and the ORF of the pestivirus genome is believed to be unstructured.

The authentic HCV initiation codon resides in the terminal loop of the RNA hairpin structure in the HCV IRES dIV. This structure is unlikely to be tolerated in the RNA binding cleft of the ribosome while the initiation codon occupies the ribosomal P site [17-19], and the ribosomal toeprint on the HCV genome at +15 from the start codon is characteristic of a eukaryotic ribosome bound to unstructured mRNA [20]. Consequently, increased stability of the HCV IRES dIV structure predictably decreases the efficiency of HCV IRES-dependent translation [17]. In addition, the HCV IRES dIV structure must presumably melt to allow the N-terminus of the HCV polyprotein to be translated from the codons in the 5' region of the ORF that are involved in the HCV IRES dIV RNA structure. The apparent requirement that the HCV IRES dIV structure unwinds to allow translation initiation suggests that its presumed viral function occurs either before the ribosome has been recruited to the mRNA or after the translating ribosome has exited the start site, which is consistent with a regulatory RNA element.

The IRE is an example of a translation regulatory RNA element that does not otherwise actively participate in the translation initiation mechanism [9]. The interaction between the IRE and its cognate binding partner, an iron regulatory protein (IRP1 or IRP2), either inhibits gene expression by inhibiting 40S ribosomal subunit binding or enhances gene expression by protecting the mRNA from nuclease degradation [21], depending on where the IRE is located in the mRNA. To inhibit ribosomal binding, the IRE must reside within the 40S ribosomal subunit binding site near the 5' cap structure. The IRE cannot bind to the 40S ribosomal subunit while it is bound to an IRP, but it has little effect on the translation initiation efficiency when it is not bound by an IRP. The active IRP concentration is controlled by the intracellular iron concentration. IRP1 is an aconitase enzyme conformer that is formed when the intracellular iron concentration is insufficient to form the characteristic aconitase ironsulfur cluster. IRP2 is homologous to IRP1 but does not form the aconitase iron-sulfur cluster [22]. Like the HCV IRES dIV structure, the IRE must melt to allow the mRNA to occupy the ribosomal RNA binding cleft.

**Figure 2:**
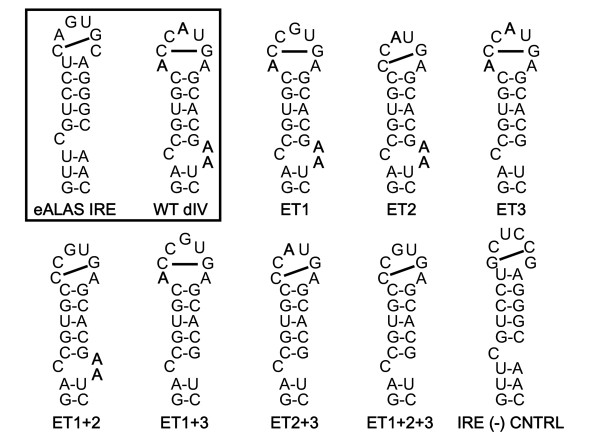
**RNA sequences and secondary structures**. Sequences and predicted secondary structures of the RNA panel evaluated in this study. The nucleotides in bold in each construct represent deviations from the consensus C-bulge IRE. Inset: the eALAS IRE and wild type HCV IRES dIV RNA are boxed for comparison.

The HCV IRES dIV RNA structure shares many of the IRE consensus structural characteristics[23,24], most closely resembling the human erythroidspecific δ-aminolevulinic acid synthase (eALAS) IRE [25] (figure 2, boxed inset). The eALAS IRE stem contains an unpaired C residue in the 5' arm (C-bulge) that is separated from the terminal loop by 5 base pairs. The C-bulge is implicated in a sequence-specific binding interaction with the IRP [26]. A prominent feature of the IRE terminal loop is an intraloop C-G base pair, which is required for its interaction with an IRP. The C-G intraloop base pair solvent-exposes the guanine base at the center of the resultant terminal tri-loop, which is also required for sequence-specific high-affinity binding to the IRP [26]. The consensus sequence of the IRE terminal loop consists of six bases, the first five of which are usually CAGUG. The sixth base can be any nucleotide, so long as it cannot base pair with C. The HCV IRES dIV RNA hairpin incorporates similar features, including a C-containing bulge in 5' arm of the stem that is separated from the terminal loop by five base pairs and the potential to form an intraloop C-G pair. In fact, the HCV IRES dIV stem differs from the consensus C-bulge IRE in only three significant ways (figure 2, boxed inset). It has an extra adenosine residue in the terminal loop, two extra adenosine residues in the 3' arm of the stem directly across from the unpaired cytosine residue, and an adenosine residue instead of a guanosine residue at the apex of the putative tri-loop formed by the intraloop C-G base pair. A mutant HCV IRES dIV structure in which all three differences are reconciled would fit the consensus definition of an authentic IRE [23,24] and would be expected to bind to an IRP with wild type affinity.

If HCV were to utilize an IRE-like mechanism to control viral expression, the RNA structure used for this regulation must be positioned within the 40S ribosomal subunit binding site. In sharp contrast to normal eukaryotic cap-dependent translation, the HCV IRES-dependent translation initiation mechanism requires that the 40S ribosomal subunit binds directly to the HCV genomic RNA sequence flanking the 5' UTR-ORF boundary to allow the start codon to occupy the ribosomal P site [20]. This mechanism would force at least part of any putative IRE-like RNA element into the ORF. Thus the HCV IRES dIV structure is properly positioned in the HCV mRNA to function as an IRE-like RNA element.

## Results

### RNA binding to hIRP1

Since the HCV IRES dIV structure differs from the consensus IRE at only three characteristics, a mutant RNA panel was constructed to evaluate the relative binding contribution of each characteristic. The panel systematically mutated the wild type HCV IRES dIV structure to a consensus IRE structure (figure 2).

**Figure 3:**
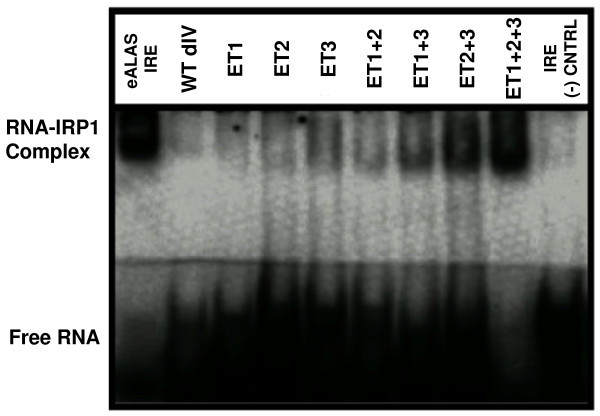
**Relative RNA – hIRP1 binding interactions**. EMSA showing the relative binding ability of hIRP1 to the RNA panel shown in figure 2.

Electrophoretic mobility shift assays (EMSA) were used to detect binding interactions between hIRP1 and the RNA species shown. Although a 100 ng concentration of hIRP1 was reported to bind to the wild type IRE [22], only the eALAS IRE and ET1+2+3 bound to hIRP1 at this concentration in our experiments (data not shown). Six of the mutant RNA sequences (ET1+2, ET3, ET2, ET1+3, ET2+3, ET1+2+3) and the eALAS IRE detectably bound to hIRP1 at an hIRP1 concentration of 240 ng (figure 3). No detectable binding to hIRP1 was observed for the wild type HCV IRES dIV, ET1, ET2, or the negative control IRE. These experiments yielded the following binding trend:

(eALAS IRE ≈ ET1+2+3) > ET2+3 > ET1+3 > ET3 > (ET2 ≈ ET1+2) > (ET1 ≈ HCV IRES dIV ≈ IRE (-) control)

### Estimate of the dissociation constant, K_d_

**Figure 4:**
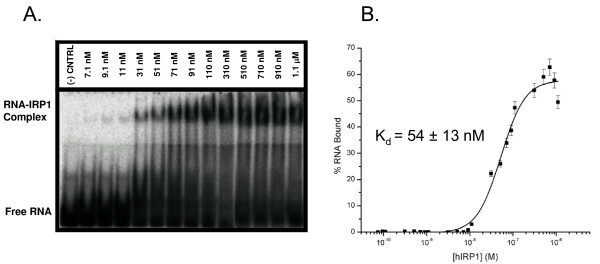
**ET2+3 – hIRP1 binding interaction concentration dependence**. (A) EMSA showing the hIRP1 concentration dependence on binding to ET2+3. Lanes showing hIRP1 concentrations of 74 pM-5.1 nM were omitted for clarity. (B) Plotted data used to determine the K_d _for the IRP1-ET2+3 interaction.

EMSA was used quantitatively to estimate the dissociation constants (*K*_*d*_) of the hIRP1-ET2+3 and hIRP1-ET1+2+3 complexes. Unsurprisingly, ET1+2+3, which adheres to the consensus definition of an IRE [23,24], bound to hIRP1 with approximately wild type affinity (*K*_*d *_~ 50 pM, data not shown). ET2+3 bound to hIRP1 with a *K*_*d *_= 54 ± 13 nM (figure 4B), which is about three orders of magnitude weaker than the consensus IRE.

## Discussion

The HCV IRES has been described as an RNA structural element that regulates viral translation. Strictly speaking, however, the HCV IRES mechanism describes the initiation of translation, not necessarily its regulation. The HCV IRES domains I-III have been well characterized in recent years, and much is known about the roles of these structures in the recruitment and assembly of the eukaryotic ribosome on the HCV RNA genome. The role of domain IV, however, remains enigmatic. It is not required for efficient initiation and is not present in the closely related pestivirus IRES, yet it is conserved across all HCV genotypes. Mutational analysis of this structure demonstrated only a modest relationship between HCV IRES-dependent translational efficiency and structural stability [17]. This observation might simply reflect the effect of RNA structure near the start codon, and does not necessarily imply a role in translation initiation. These observations are consistent with a regulatory RNA element that modulates translation efficiency in response to an appropriate biological signal but does not otherwise participate directly in the recruitment and assembly of the ribosome. The IRE-IRP mechanism represents a useful model for this type of interaction.

A cursory comparison of the structure and genomic location of the HCV IRES dIV RNA structure to the C-bulge eALAS IRE is provocative and suggests a correlated similarity of function. The wild type HCV IRES dIV RNA does not measurably bind to hIRP1, however, demonstrating that it is not an authentic IRE. The ET1+2+3 RNA, which adheres to the consensus definition of an IRE, bound to IRP1 with wild type efficiency as expected. Deleting the two A residues in the bulge of the wild type HCV IRES dIV RNA (the ET3 effect) contributed the greatest relative effect on binding affinity, followed by deletion of an A residue in the terminal loop (the ET2 effect), suggesting the importance of the correct three dimensional RNA structure on hIRP1 binding. The G residue at the apex of the terminal tri-loop and C-bulge residue are conserved in the consensus IRE and are presumably required for sequence-specific interactions between the IRE and recognition motifs on the IRP [9]. These residues are displayed in three dimensions relative to each other by the structural features of the stem and the terminal loop. Altering the IRE structure likely misaligns the recognition nucleotides with their respective recognition motifs on hIRP1. A well-defined and rigid IRE binding pocket would not accept a stem of the wrong shape, regardless of the sequential positions of the recognition nucleotides. The ET2 and ET3 effects are consistent with the reported IRE characteristics required for efficient IRE-IRP interaction [27]. Mutating the A residue of the start codon to a G residue (the ET1 effect), a sequence-only mutation, showed the least effect on hIRP1 binding. The ET1 effect was unexpected, since the literature reports that the G residue in the terminal loop is essential [28]. Either purine residue in this position yielded significant IRP1 binding in our hands, with only a slight preference for G (compare ET2+3 to ET1+2+3 in figure 3).

## Conclusion

The primary conclusion from this work is that the wild type HCV IRES dIV RNA structure is not an authentic IRE, as it does not bind appreciably to the recombinant IRP1 protein. The hypothesis that HCV utilizes this structure to control viral expression by an IRE-like mechanism remains viable, although a putative cellular or viral factor that fulfils the analogous IRP function must be identified to properly evaluate this hypothesis. The p85 and p100 proteins that have been recently shown to bind to both the HCV IRES and the iron responsive element with high affinity [6] could serve this purpose. For now, the role of the highly conserved HCV IRES dIV structure remains unresolved.

## Methods

### Expression and purification of human IRP1

Human IRP1 (hIRP1) fused to glutathione S-transferase (GST) was expressed and purified as previously described [22] with some modifications. The pGEX-2T plasmid (Dr. Lukas Kuhn, ISREC, Epalinges, Switzerland) was transformed into HB101 cells and hIRP1 expression was induced with 0.1 or 0.5 mM IPTG (Sigma-Aldrich, St Louis, MO) overnight at room temperature. Each lysis reaction contained 2 mL (approximately 0.2 g) of cell lysate, 10 μL of protease cocktail inhibitor (Sigma-Aldrich, St Louis, MO) and 15,000 U of lysozyme (Novagen, San Diego, CA). The cells were lysed by sonication in PBS buffer (150 mM NaCl, 16 mM Na_2_HPO_4_, 4 mM NaH_2_PO_4_, pH 7.3) containing 1% triton-X-100 (Sigma-Aldrich, St Louis, MO). The cell lysate was then incubated at 4°C for 30 minutes and spun at 10,000 rpm for 30 minutes. hIRP1 was purified on a 50% glutathione sepharose resin column (Amersham, Piscataway, NJ). The protein was eluted with 50 mM Tris and 10 mM reduced glutathione (pH 8.0), concentrated using microcon YM 50 (Millipore, Jaffrey, NH) and its concentration, 1.2 μg/μL, was determined using the Micro Lowry method (Sigma-Aldrich, St Louis, MO). Purified and unpurified hIRP1 was visualized on an SDS-PAGE and confirmed by Western blot using rabbit anti-rat IRP1 polyclonal antibodies (Alpha Diagnostic, San Antonio, TX).

### RNA synthesis

RNA was synthesized by *in vitro *transcription from double-stranded DNA oligonucleotides[29,30]. Seven pairs of DNA mutant oligonucleotides corresponding to RNA sequences (ET1, ET2, ET3, ET1+2, ET1+3, ET2+3, and ET1+2+3) were derived from wild type genotype 1b HCV dIV RNA (figure 2). The sequences of the cDNA oligonucleotides representing the wild type HCV dIV RNA (HCV nt 331–354), the seven RNA mutants, a positive control IRE (erythroid δ-aminolevulinate synthase IRE (eALAS IRE)), and a negative control IRE[31], all fused downstream of a T7 bacteriophage promoter were synthesized and purified by PAGE (IDT, Coralville, IA). Complementary oligonucleotides (76 μM each) were annealed in annealing buffer (10 mM MgCl_2_, 200 mM Tris-HCl, pH 8) at 95°C for 3 minutes, and cooled to room temperature. RNA was transcribed *in vitro *from 456 nM of each annealed DNA using T7 MAXIscipt (Ambion Inc. Austin, Texas) following the manufacturer's instructions except that α-^32^P CTP (800 Ci/mmole) (MP Biologicals, Irvine, CA) and 12.5 U of RNase Inhibitor (Ambion Inc. Austin, Texas) were added. The reactions were incubated at 37°C for 1 hour, treated with DNase for 25 minutes, quenched by the addition of 25 nM EDTA (Ambion Inc. Austin, Texas), and the RNA transcripts were purified on a 20% denaturing PAGE gel.

### Electrophoretic mobility shift assays

EMSAs were used to detect and visualize binding interactions between hIRP1 and the oligoribonucleotides *in vitro*. Each RNA transcript (0.3 ng, approximately 3.0 × 10^5 ^cpm) was folded at 75°C in 20 mM MgCl_2 _for 2 minutes and was cooled to room temperature. hIRP1 (with GST-tag) was activated with 2% β-mercaptoethanol prior to use. Binding reactions consisted of 0.3 ng of each folded RNA transcript and 240 ng of hIRP1 in binding buffer (10 mM HEPES pH 7.6, 2 mM MgCl_2_, 40 mM KCl, 5% glycerol, and 1 mM DTT) to a 20 μL total volume. Reaction mixtures were incubated at room temperature for 30 minutes. Heparin (0.63 μg/μL) was then added, and the mixture was incubated at room temperature for 10 additional minutes. RNA-protein complexes were resolved on a discontinuous native polyacrylamide gel (7% top and 14% bottom) in 0.5× TBE buffer to allow both the free and IRP1-bound RNA to be visualized on the same gel. The gel was dried, exposed to a phosphor-imager plate (Molecular Dynamics) overnight. The bands were visualized on a Storm phosphorimager (Molecular Dynamics) and quantified using ImageQuaNT software (Molecular Dynamics).

EMSA was used quantitatively to estimate the dissociation constants (*K*_*d*_) of the hIRP1-ET2+3 and hIRP1-ET1+2+3 complexes. Activated hIRP1 was serially diluted in binding buffer (10 mM HEPES pH 7.6, 2 mM MgCl_2_, 40 mM KCl, 5% glycerol, and 1 mM DTT) to give a final concentration range of 74 pM to 1.1 μM. Diluted hIRP1 was incubated with 5.2 nM α^32^P-CTP labeled RNA. Complexed and free RNA species were resolved on a discontinuous native gel (figure 4A), visualized and quantified as previously described. The reported *K*_*d *_is an average of three independent experiments and was calculated by nonlinear curve fit using the Origin program (MicroCal)[32,33] (figure 4B). Calculations assumed that the plateau in the curve represents complete RNA binding and that there was only one hIRP1 binding site on the RNA. To allow for the possibility that the hIRP1-RNA complexes might have dissociated during resolution on the native gel, the complex bands were quantified to include all radioactivity that ran ahead of these complexes with respect to the control lane lacking hIRP1.

## Competing interests

The authors declare that they have no competing interests.

## Authors' contributions

ET synthesized and purified the RNA and hIRP1, carried out the EMSA experiments, and helped draft the manuscript. JMP reproduced and verified the results. WBL conceived of the study, participated in its design and coordination, and drafted the manuscript. All authors read and approved the final manuscript.
